# Tuning Mechanical Characteristics and Permeability of Alginate Hydrogel by Polyvinyl Alcohol and Deep Eutectic Solvent Addition

**DOI:** 10.3390/bioengineering11040371

**Published:** 2024-04-12

**Authors:** Tadej Menegatti, Tilen Kopač, Polona Žnidaršič-Plazl

**Affiliations:** 1 Faculty of Chemistry and Chemical Technology, University of Ljubljana, Večna pot 113, SI-1000 Ljubljana, Slovenia; tadej.menegatti@fkkt.uni-lj.si (T.M.); tilen.kopac@fkkt.uni-lj.si (T.K.); 2 Chair of Micro Process Engineering and Technology—COMPETE, University of Ljubljana, Večna pot 113, SI-1000 Ljubljana, Slovenia

**Keywords:** hydrogel, deep eutectic solvent, polyvinyl alcohol, alginate, rheology, permeability

## Abstract

Alginate-based hydrogels are widely utilized for various applications, including enzyme immobilization and the development of drug delivery systems, owing to their advantageous characteristics, such as low toxicity, high availability and cost-effectiveness. However, the broad applicability of alginate hydrogels is hindered by their limited mechanical and chemical stability, as well as their poor permeability to hydrophobic molecules. In this study, we addressed the mechanical properties and chemical resistance of alginate hydrogels in a high-pKa environment by the copolymerization of alginate with polyvinyl alcohol (PVA). The addition of PVA resulted in a threefold improvement in the shear modulus of the copolymeric hydrogel, as well as enhanced chemical resistance to (*S*)-α-methylbenzylamine, a model molecule with a high pKa value. Furthermore, we addressed the permeability challenge by introducing a betaine–propylene glycol deep eutectic solvent (DES) into the PVA-alginate copolymer. This led to an increased permeability for ethyl 3-oxobutanoate, a model molecule used for bioreduction to chiral alcohols. Moreover, the addition of the DES resulted in a notable improvement of the shear modulus of the resulting hydrogel. This dual effect highlights the role of the DES in achieving the desired improvement of the hydrogel as an immobilization carrier.

## 1. Introduction

Biocatalysis, acknowledged as a foundational element for sustainable production and circular economy, has been identified as a key green engineering area in pharmaceutical and fine chemical production alongside continuous processing [1]. Continuous biocatalytic processes, facilitated by the immobilization of biocatalysts that retain enzymes within the reactor, play a pivotal role in propelling the advancements in biocatalysis [2], drug/gene delivery [3] and biosensing [4].

Despite their advantages, continuous processes face challenges such as low conversions and enzyme leakage over extended operational periods, which hinder their efficacy. These challenges can stem from changes in enzyme structure during immobilization on various supports, which is the most common but also a costly approach [2]. Alternatively, entrapping enzymes in porous structures through the crosslinking of various (bio)polymers offers a more cost-effective and non-invasive immobilization technique [5,6].

Alginate, an anionic polysaccharide derived from seaweeds, is widely employed for the immobilization of biocatalysts, leading to the formation of hydrogels in the presence of multivalent cations like Ca^2+^, Ba^2+^, or Fe^3+^ [7]. However, calcium alginate hydrogels encounter challenges, notably instability in solutions with a pH higher than the pKa of their substituent groups. This limitation restricts their applicability in specific biotransformations [8]. Similarly, PVA hydrogels, especially those obtained through the freeze–thawing technique, encounter issues related to enzyme leakage and stability [9]. Addressing these challenges, a copolymer hydrogel that combines PVA and alginate has been developed, demonstrating improved stability and enhanced suitability for biocatalyst immobilization [10,11].

To achieve a comprehensive understanding of hydrogel structures, rheological characterization offers valuable insights into critical parameters such as pore size and shear modulus [12]. These parameters play a vital role in elucidating enzyme immobilization mechanisms and addressing challenges associated with enzyme leakage and stability within hydrogel structures. Moreover, hydrogels pose a challenge in terms of the low diffusivity and permeability of molecules with low water solubility, limiting their effectiveness in various applications [13]. The incorporation of deep eutectic solvents (DESs), comprising two or more components that form a eutectic mixture with distinctive properties, introduces a novel strategy to improve the performance of hydrogels. This enhancement expands their applicability and effectiveness across various domains [14,15]. The reduction in the melting point of DESs is commonly ascribed to the establishment of hydrogen bonding interactions among the constituent compounds. DESs have attracted significant attention due to their ability to solubilize lipophilic molecules, making them valuable in extraction and dissolution applications. They have been recognized as “solvents for the 21st century” and have shown promise in biocatalytic reactions [16]. DESs offer remarkable tunability by adjusting the hydrogen bond acceptors and donors, as also noted in recent research [17]. This flexibility underpins their wide range of applications. Additionally, DESs exhibit low toxicity, rendering them suitable for environmentally sensitive processes and applications prioritizing human safety [18].

With their dual nature of lipophilicity and hydrophilicity, DESs play a distinctive role in enhancing the permeability of lipophilic molecules through hydrogels [19]. To effectively utilize biocatalysts with hydrophobic substrates, it is essential to chemically modify immobilization carriers. This modification aims to alter surface properties by enhancing hydrophobicity or introducing amphiphilic characteristics to improve the availability of the substrate for the immobilized biocatalyst [20].

For example, the encapsulation of curcumin in alginate–chitosan hydrogel beads using a DES composed of choline chloride and glycerol led to a substantial increase in curcumin solubility compared to phosphate-buffered saline. This enhanced solubility in the DES is attributed to robust molecular interactions, including hydrogen and van der Waals forces [13]. Another study demonstrated that the addition of a choline chloride and mannose DES to PVA nanofibers enhanced their fast-dissolving properties in artificial saliva solutions, indicating potential for fast drug delivery in oral cavities [21]. Recently, Sun et al. [15] constructed a chitosan-based supramolecular aerogel through hydrogen bonding between chitosan, a natural DES comprising glycerol and L-lactic acid, and PVA. This aerogel exhibited a well-defined skeleton-type 3D network structure with lower density and higher porosity, along with excellent water absorption capability [22]. Although DESs have primarily been utilized in hydrogel preparation for drug delivery [13,19], extraction purposes [22] or medical dressings [15], their role as solvents in biocatalytic processes has also gained increased attention in recent years [14].

In this study, our main objective was to optimize the mechanical and chemical properties of hydrogels by incorporating PVA into sodium alginate hydrogels, which we previously investigated for enzyme immobilization [11,23]. We assessed the chemical resistance of the resulting hydrogel in environments with a high pKa value using (*S*)-α-methylbenzylamine, a typical amine donor in transamination reactions employing amine transaminases [11]. Subsequently, we introduced a portion of the selected DES based on betaine and propylene glycol into the resulting copolymeric mixture to enhance its permeability for ethyl 3-oxobutanoate, a model substrate that is used for biocatalytic reduction to chiral alcohols [24]. This approach had the primarily aim of enhancing permeability, addressing challenges associated with low-water-solubility of molecules used in biotransformations and thereby expanding the potential applications of the hydrogel as an immobilization carrier in biocatalytic processes.

## 2. Materials and Methods

### 2.1. Materials

Sodium alginate, phenylboronic acid, (*S*)-α-methylbenzylamine (MBA), ethyl 3-oxobutanoate and 4-(2-hydroxyethyl)-1-piperazineethanesulfonic acid (HEPES) were all purchased from Sigma Aldrich (St. Louis, MO, USA), CaCl_2_ was from Carlo Erba reagents (Milan, Italy), and polyvinyl alcohol (PVA, MW = 13,000–23,000 Da) was purchased from Acros organics (Morris Plains, NJ, USA). The DES components propylene glycol and betaine were from Alkaloid (Skopje, North Macedonia) and Sigma Aldrich (St. Louis, MO, USA), respectively.

### 2.2. DES Preparation

The deep eutectic solvent (DES) solution was prepared by combining betaine and propylene glycol in a molar ratio of 1:3. Water was added to the mixture to achieve a 50% (*v*/*v*) solution. The resulting blend was heated to 80 °C, thoroughly mixed, and then allowed to cool to room temperature.

### 2.3. Hydrogel Preparation and Its Water Content Assessment

A 2% (*w*/*v*) sodium alginate solution was obtained by dissolving sodium alginate in demineralized water through heating and mixing, followed by cooling to room temperature. The resulting solution was poured into a circular mold, and a 2% (*w*/*v*) CaCl_2_ solution was sprayed on top to initiate the crosslinking process. After 1 h, crosslinking was halted, and the produced calcium alginate was washed with demineralized water and stored until further analysis.

Copolymeric hydrogels were prepared by combining 2% (*w*/*v*) sodium alginate with varying portions of PVA in demineralized water. The mixture was heated to 80 °C to dissolve PVA, and after cooling to room temperature the copolymeric hydrogel was generated using the same crosslinking process as with the alginate hydrogel. The crosslinking solution for PVA chains included 2% (*w*/*v*) phenylboronic acid (PBA) to initiate crosslinking via boron ions. Details on the prepared hydrogels are presented in Table 1.

A DES-incorporated hydrogel was prepared by substituting water with the DES in the copolymeric mixture before the crosslinking process. In this instance, the DES was formulated by combining betaine and propylene glycol in a molar ratio of 1:3 and subsequently diluting with 50% water, as outlined in Section 2.2. Specifically, the 50% DES solution was further diluted with water and introduced into the PVA-alginate mixture containing 8% (*w*/*v*) PVA to yield a final DES concentration of 10 to 30%. The crosslinking procedure to obtain hydrogels followed the same method as described previously. Detailed information regarding these modified hydrogels is provided in Table 2. Furthermore, samples with soley 20% (*w*/*v*) betaine and soley 20% (*w*/*v*) PEG addition were made (abbreviated 8-2-BET and 8-2-PEG) to check their shear modulus values.

The water content in hydrogels with or without DES addition was evaluated using a high-performance HR83 moisture analyzer (Mettler Toledo, Columbus, OH, USA). The excess water was first removed from the outer surface using a wipe tissue and then the sample was placed in the moisture analyzer. The sample was dried at a set temperature of 80 °C until no mass change was recorded. The water content was calculated from the initial weight of the non-dried sample.

### 2.4. Hydrogel Chemical Stability Assessment

The examination of chemical stability involved immersing the prepared hydrogels in a 50 mM solution of (*S*)-α-methylbenzylamine within HEPES buffer. Following varying incubation times, the hydrogels were removed from the solution and rinsed with demineralized water before subsequent rheological measurements. The hydrogel compositions chosen for the assessment of chemical stability in this alkaline environment were Alg and 8-2, as detailed in Table 1.

### 2.5. Rheological Measurements

Initially, amplitude sweep tests were conducted using a Physica MCR 301 rheometer (Anton Paar, Graz, Austria) equipped with a 50 mm diameter crosshatched plate (PP50/P2) to identify the linear viscoelastic region of the hydrogels. Subsequently, frequency-sweep tests were carried out at a constant strain (0.1%), varying oscillation frequencies from 100 to 0.01 Hz at 25 °C. The experimental data were described using the generalized Maxwell model, comprising the equilibrium modulus (*G_e_*) and Maxwell elements (*G_i_*) as relaxation modules. The equations for the frequency-dependent viscoelastic moduli in the generalized Maxwell model are as follows:

(1) $$G^{\prime }=G_{e}+\sum_{i=1}^{n}\frac{G_{i}\lambda_{i}^{2}\omega^{2}}{1+\lambda_{i}^{2}\omega^{2}}$$

(2) $$G^{\prime \prime }=\sum_{i=1}^{n}\frac{G_{i}\lambda_{i}\omega }{1+\lambda_{i}^{2}\omega^{2}}$$

Here, *λ_i_* represents the relaxation time of the *i*th Maxwell element, *n* is the number of considered Maxwell elements and *ω* is the frequency.

The obtained mechanical spectra for *G*′ and *G*″ were then employed to determine the shear modulus (*G*) and the average mesh (pore) size (*ε*). The shear modulus is estimated as the sum of Maxwell’s elastic elements *G_e_* and *G_i_*:

(3) $$G=\sum_{i=1}^{n}G_{e}+G_{i}$$

The average mesh (pore) size (*ε*) was subsequently estimated using Flory’s theory:

(4) $$\varepsilon_{rheo}=\sqrt[{3}]{\frac{6RT}{\pi G{\left(\frac{\varphi_{p}}{\varphi_{p0}}\right)}^{2/3\;N_{A}}}}$$

In this equation, *N_A_* is the Avogadro number, *R* is the gas constant, *T* is the temperature in K and *φ_p_*_0_ and *φ_p_* are polymer volume fractions under crosslinked conditions and rheological measurement conditions, respectively [12].

### 2.6. Permeability Assessment for DES-Infused Hydrogel

**Figure 1:**
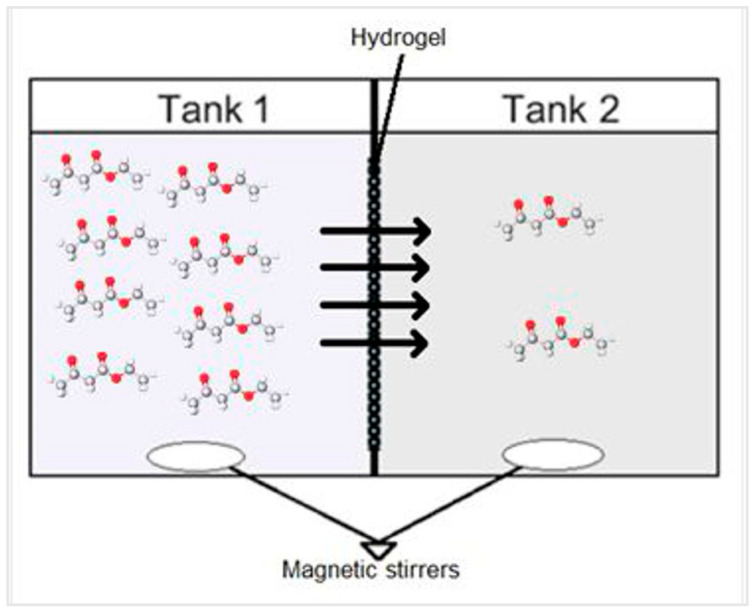
Schematic of the custom-made two-tank cell for diffusion coefficient assessment.

The permeability of ethyl 3-oxobutanoate molecules through the porous copolymeric hydrogel was investigated at room temperature using a custom-built two-tank cell, which is depicted in Figure 1. In this setup, a hydrogel layer served as a separator between the two solutions. Tank 1 contained the solution with ethyl 3-oxobutanoate molecules, while tank 2 held the 50% DES solution. The hydrogel film functioned as a barrier, allowing ethyl 3-oxobutanoate molecules to diffuse through the pores to the opposite side and vice versa. Samples from both solutions were collected at different time intervals for subsequent analysis. It is noteworthy that the measured thickness value was employed in diffusion coefficient calculations.

The mathematical representation of transport through the hydrogel is based on a simplified and linearized derivation of Fick’s second law of diffusion (Equation (5)) [25]:

(5) $$D_{g}=\frac{1}{\beta \cdot t}\ln\left(\frac{C_{1}(t)-C_{2}(t)}{C_{0}^{1}-C_{0}^{2}}\right)$$

with

(6) $$\beta =\left(\frac{A_{H}}{W_{H}}\right)\cdot \left(\frac{1}{V_{1}}+\frac{1}{V_{2}}\right)$$

where *C*_1_(*t*) and *C*_2_(*t*) represent the ethyl 3-oxobutanoate concentrations in tank 1 and tank 2 at time *t*, respectively, and $C_{0}^{1}$ and $C_{0}^{2}$ represent the initial concentrations of ethyl 3-oxobutanoate in tank 1 and tank 2, respectively. *A_H_* represents the effective cross-sectional area of diffusion in the hydrogel sample and *W_H_* represents the width (thickness) of the hydrogel sample. *V*_1_ and *V*_2_ represent the volume of solutions in tank 1 and tank 2, respectively. In tank 1, ethyl 3-oxobutanoate was dissolved in 50% DES, while in tank 2 no ethyl 3-oxobutanoate was added to the 50% DES solution.

The thickness of the hydrogel layers specified in Section 3.4 was measured at multiple points using a Digimatic height gauge (Mitutoyo, Kawasaki, Japan), and the average value was subsequently calculated.

### 2.7. Hydrogel Stability in 50% DES

The stability of the hydrogel in a 50% DES solution was evaluated by preparing samples according to the procedure outlined in Section 2.3 and immersing them in the 50% DES solution at room temperature. Rheological analysis was performed on days 7 and 14, in addition to an initial assessment of the shear modulus as outlined in Section 2.5. Relative changes in *G* were calculated with respect to the initial *G* values.

### 2.8. FTIR Analysis

FTIR analysis was conducted using IRTracer-100 instrument (Shimadzu, Kyoto, Japan), with hydrogel samples dried in a vacuum prior to analysis to ensure the absence of water. The DES was measured without the addition of water.

### 2.9. Zeta Potential Analysis

Zeta potential analysis of the copolymer solutions was performed on a Litesizer 500 (Anton Paar, Graz, Austria) at 25 °C, taking 5 measurements of 100 runs with Smoluchowski approximation used in the calculations.

Zeta potential analyses were conducted on diluted aqueous solutions of non-crosslinked polymers, namely 2% alginate, 10% PVA, and copolymers such as 4-2, 8-2 and 8-2-30% DES. To ensure precise measurements, the solutions were diluted with demineralized water by a factor of ten to effectively reduce viscosity while maintaining accuracy.

### 2.10. Ethyl 3-Oxobutanoate Analysis

The solutions of ethyl 3-oxobutanoate were quantitatively analyzed using a UV/Vis spectrophotometer (Shimadzu, Kyoto, Japan) at a wavelength of 245 nm to determine the concentration of ethyl 3-oxobutanoate in each chamber over time. The diffusion coefficient, *D_g_*, was calculated from these results using Equations (5) and (6).

## 3. Results and Discussion

As demonstrated in our prior studies, the introduction of PVA into the alginate hydrogel matrix resulted in a copolymeric hydrogel with excellent ability to retain both yeast cells [10] and purified amine transaminase [11]. In both investigations, biocatalysts were effectively immobilized within a copolymeric hydrogel and embedded in a microreactor, showcasing sustained productivity without significant deterioration over an extended period. In this study, additional rheological assessments were undertaken to determine the shear modulus (*G*) and the average pore size (*ε*). These supplementary analyses aimed to provide further insight into the impact of incorporating PVA into the alginate hydrogel, with the objective of studying its performance as an immobilization matrix/carrier.

### 3.1. PVA’s Effect on Shear Modulus and Average Pore Size

**Figure 2:**
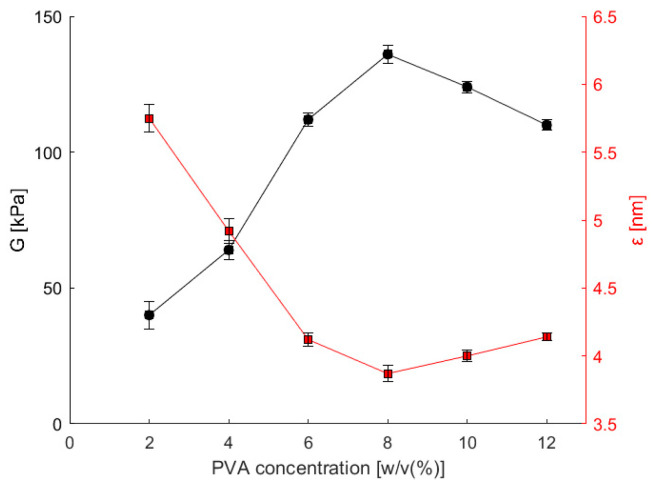
The effect of PVA concentration in a copolymeric hydrogel on the estimated shear modulus *G* and the average pore size *ε*. Error bars indicate standard deviations of triplicates (*n* = 3).

Hydrogels with various compositions, as specified in Table 1, underwent the rheological measurements detailed in Section 2.5, and the shear modulus (*G*) and average pore size (*ε*) were calculated using Equations (1)–(4). Figure 2 illustrates the dependence of both variables on PVA concentration in the prepared copolymeric hydrogel. The results show a noticeable increase in the shear modulus with an escalating PVA content in the copolymer hydrogel. In particular, the shear modulus surpassed the 136 kPa threshold at 8% PVA, marking a threefold augmentation compared to the 2% alginate hydrogel without PVA addition. Interestingly, a subsequent increase in PVA content to 12% resulted in a reduction of the shear modulus to 110 kPa.

Considering the interdependence between shear modulus and pore size, it becomes evident that at 8% PVA, the average pore size (*ε*) diminished to below 4 nm. This renders the hydrogel with 8% PVA (8-2 hydrogel) the most suitable for the successful immobilization of enzymes larger than the pore size, as was the case with the amine transaminase used in our previous study [11].

### 3.2. PVA’s Effect on Chemical Stability of Copolymeric Hydrogel in Amine Solution

To assess the chemical stability of the prepared hydrogels, they were immersed in a 50 mM (*S*)-α-methylbenzylamine aqueous solution. This amine, characterized by a pKa of 9.04 ± 0.10, is commonly employed as an amine donor in biotransformations involving amine transaminases [11,26]. It was also previously documented that alginate hydrogels exposed to a basic environment undergo the deprotonation of carboxyl groups due to hydroxide ions, which cause repulsion and hence are responsible for swelling [8].

**Figure 3:**
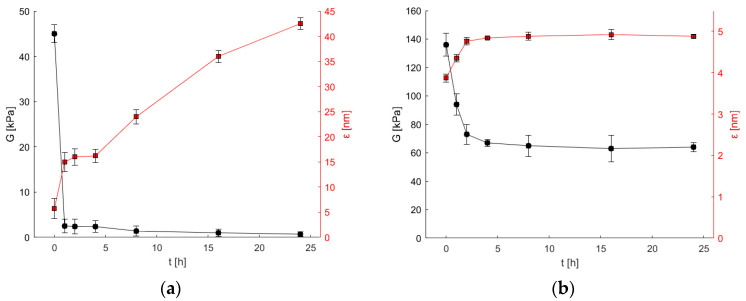
Shear modulus *G* and the average pore size *ε* as a function of the incubation time with 50 mM (*S*)-α-methylbenzylamine for (**a**) Alg and (**b**) 8-2 hydrogels. Error bars indicate the standard deviations of triplicates (*n* = 3).

Hydrogels with various compositions, specifically Alg and 8-2 as detailed in Table 1, were exposed to 50 mM (*S*)-α-methylbenzylamine for different periods and subjected to further rheological characterization. In Figure 3a, which illustrates the time dependence of the calculated shear modulus (*G*) and average pore size (*ε*) for the alginate hydrogel (Alg) incubated in (*S*)-α-methylbenzylamine, rapid degradation within one hour of exposure was observed. Moreover, the average pore size increased over time, reaching a final size of 42 nm after 24 h. In the context of enzyme immobilization, this phenomenon could potentially lead to complete enzyme leakage from the hydrogel structure.

In contrast, Figure 3b, which illustrates the time dependence of the calculated shear modulus (*G*) and average pore size (*ε*) for the copolymer 8-2 hydrogel with 8% (*w*/*v*) PVA incubated in (*S*)-α-methylbenzylamine, shows changes in hydrogel characteristics to a much lesser extent. Both the shear modulus and average pore size remained constant after 4 h of exposure, with the pore size staying below 5 nm, theoretically ensuring the successful retention of immobilized enzymes. After 24 h, the shear modulus remained at 50% of the initial value, exhibiting no significant decrease after 4 h of exposure (Figure 3b).

The copolymeric 8-2 hydrogel, which consisted of PVA and alginate, exhibited significantly higher chemical resistance to the (*S*)-α-methylbenzylamine solution compared to the pure alginate hydrogel (Alg). The latter lost its structural integrity after only one hour of exposure, confirming the original hypothesis that the addition of PVA leads to hydrogels that are better suited as matrices for enzyme immobilization.

We can hypothesize that pH significantly affects the behavior of alginate polymer chains. The acidic nature of alginate arises from its mannuronic and guluronic acid residues, with reported pKa values of approximately 3.38 and 3.65, respectively, while in our previous experiments, the alginate pKa determined from the titration curve at the half-equivalence point was found to be 3.57 [8]. These pKa values indicate that alginate’s behavior in solution and its ability to form gels can be significantly influenced by the solution’s pH due to the ionization of its carboxylic groups. On the other hand, the deprotonation of carboxyl groups generates a repulsive electrostatic force between the negatively charged groups on the surface on alginate polymers that expands the polymer chains within the hydrogel network. As a consequence, water diffusion into the hydrogel increases (swelling), and a decrease in the shear modulus (which results from a decrease in crosslink density due to repulsive electrostatic interactions) for alginate hydrogels is observed in Figure 3a (black circles).

In contrast, polyvinyl alcohol (PVA) lacks a well-defined pKa value, unlike substances with strong acidic or basic functional groups. This is because PVA is a neutral synthetic polymer composed of vinyl alcohol units. It does not contain ionizable groups like carboxylic acids or amines, which are typically responsible for pKa behavior. This study concludes that the hydrogels based on PVA and alginate were prepared and investigated in their swollen state. This minimized the effect of pH on the material properties. The highly crosslinked network achieved with calcium ions (shear modulus exceeding 100 kPa in Figure 2 and Figure 3b) further restricted the hydrogels’ ability to swell, also contributing to the reduced influence of pH.

### 3.3. FTIR Analysis of DES-Infused Hydrogel Samples

**Figure 4:**
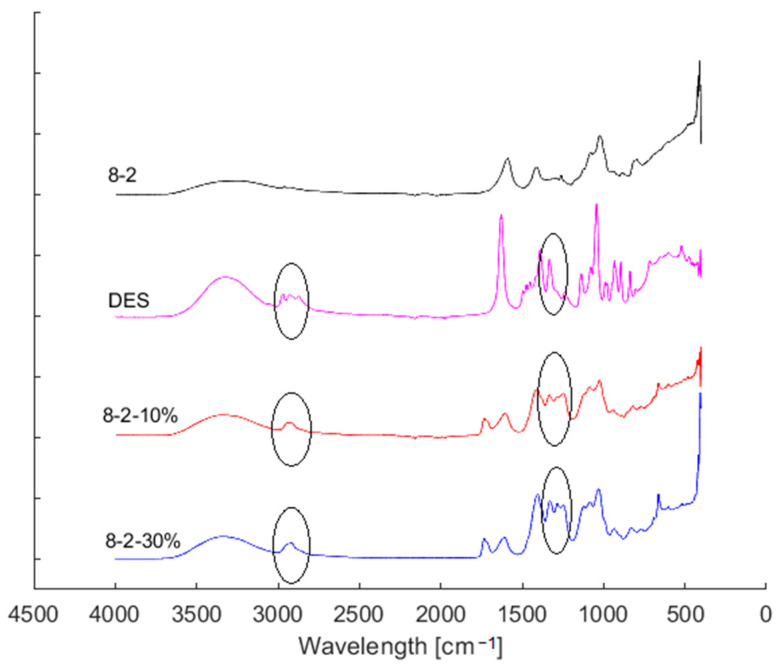
FTIR spectra of DES and different hydrogel samples with two distinctive regions (2950 cm^−1^ and 1400–1200 cm^−1^) highlighted with black circles as indications of DES’s presence in the hydrogels.

In this section, we delve into the FTIR analysis of DES-infused hydrogel samples, comparing different DES concentrations within the hydrogel matrix. Illustrated in Figure 4 are the spectra of the 8-2, 8-2-10% and 8-2-30% hydrogel samples, as well as the spectrum of pure DES. The pure DES spectrum (pink line) exhibits a main O-H stretching vibration peak at 3350 cm^−1^ and a C-H stretching vibration at 2950 cm^−1^. Both peaks are also seen in the hydrogels with added DES, indicating the presence of these C-H bonds attributable to DES. Another stretching peak from DES, not shown in the pure PVA hydrogel, is at 1300 cm^−1^, corresponding to the C=O bond from betaine.

These regions correspond to characteristic vibrational modes, suggesting the successful incorporation of DES into the hydrogel structure. Furthermore, observed peaks corresponding to PVA and alginate affirm the synthesis of the copolymeric hydrogel, enriching our understanding of its chemical composition and structural characteristics.

### 3.4. Effect of DES Addition on Mechanical Properties, Water Content and Stability of Copolymeric Hydrogels

In this study, a particular DES was chosen because of its potential application in the bioreduction of ketones, specifically ethyl 3-oxobutanoate, to chiral alcohols by yeast cells [24]. The aim was to identify a solvent that would increase the solubility of the target ketone while minimizing interference in yeast’s bioreductive activity. The DES and its molar ratio were selected based on the existing literature [27] and accounting for the solubility enhancement for ethyl 3-oxobutanoate using the conductor-like screening model COSMO-RS software (BIOVIA COSMOtherm 2020 version 20.0.0, Dassault Systèmes, Vélizy-Villacoublay, France). Following this assessment, the betaine–propylene glycol DES was chosen for further experimentation.

**Figure 5:**
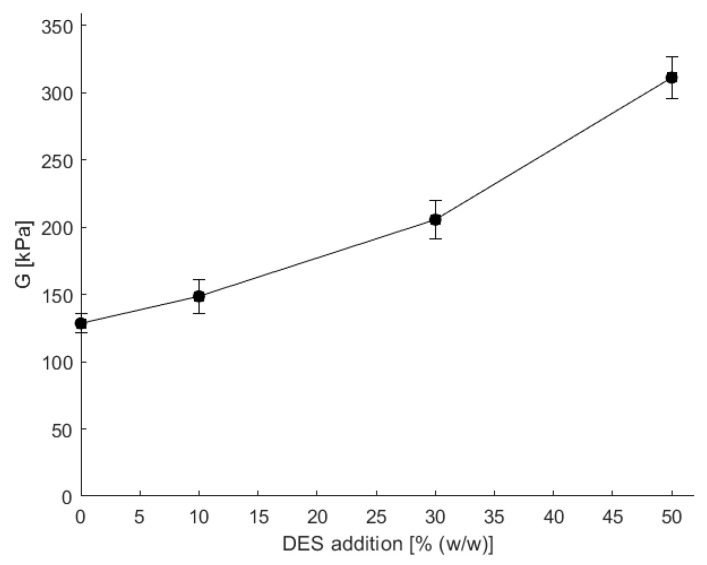
The dependence of shear modulus (*G*) on different additions of DES when preparing copolymeric hydrogels. Error bars indicate standard deviations of triplicates (*n* = 3).

The 8-2 PVA-alginate copolymeric hydrogels, prepared with or without different concentrations of betaine–propylene glycol DES, were characterized using a rheometer, and the shear modulus *G* was evaluated from the data as explained in Section 2.5. The addition of the viscous DES into the copolymeric mixture resulted in an increase in the resulting viscosity of the copolymeric mixture. After crosslinking, the shear modulus of hydrogels with DES addition was calculated, and is presented in Figure 5.

As is evident from Figure 5, the increase in DES addition, and thereby the increased viscosity, led to more mechanically robust crosslinked hydrogels, as the shear modulus increased. Additionally, the hydrogen bonding between DES components also increased the crosslinking density, where the incorporation of 50% DES in the hydrogel preparation yielded more than a twofold increase in shear modulus (from 135 kPa to 321 kPa). The pore size was not calculated since the used model does not include potential additional bonding. However, the higher viscosity of the copolymeric mixture poses challenges in handling, particularly in its role as an immobilization carrier. Additionally, the increased thickness of the hydrogel makes it difficult to achieve thin layers, which are more suitable, especially for use in flow microreactors where small diffusion layers are desirable [10,11]. Additional experimental investigations were carried out to elucidate the observed enhancement in shear modulus upon adding the selected DES. When solely betaine or polyethylene glycol (PEG) was introduced into the copolymeric solution, a reduction in shear modulus was observed relative to the base composition (8-2 hydrogel) and notably to the formulation incorporating DES at an equivalent percentage. Specifically, the shear modulus experienced a nearly 1.5-fold decrease with PEG supplementation, whereas the introduction of betaine resulted in a twofold reduction in shear modulus compared to the 8-2 hydrogel Figure S1). These findings corroborate the unique impact of DES on hydrogel properties, distinguishing it from the influence of DES constituents.

Measurements of the water content in hydrogels with and without DES addition were also performed, as described in Section 2.3. The alginate hydrogel (Alg) showed a water content of approximately 89%, while the addition of 8% PVA (8-2 hydrogel) led to a slight decrease in water content to about 83%. On the other hand, the addition of DES to various hydrogels had minimal effect on water content, with variations remaining within 1% compared to samples with the same polymer composition without DES (Table S1). These results suggest that while polymer composition affects water retention in a hydrogel, the presence of DES does not significantly alter the water content. These findings improve our understanding of the hydration properties of hydrogels for their potential applications.

**Figure 6:**
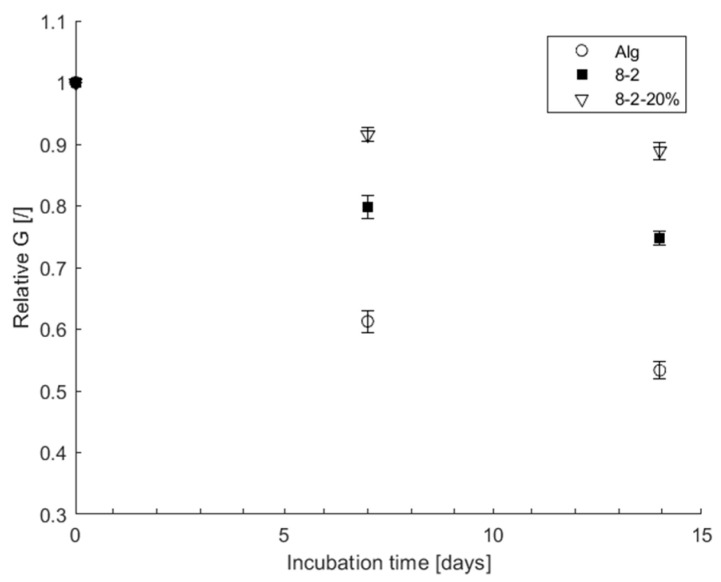
The stability of hydrogel samples when incubated in 50% DES solution as a function of shear modulus, relative to starting shear modulus. Error bars indicate the standard deviations of triplicates (*n* = 3).

When comparing the stability of Alg, 8-2 and 8-2-20% in 50% betaine–propylene glycol DES, as outlined in Section 2.7, a notable difference in structural integrity emerged. As observed in Figure 6, the hydrogel augmented with 20% DES surpassed both its pure 8-2 hydrogel counterpart and the alginate hydrogel in terms of maintaining structural integrity. This intriguing revelation suggests that the inclusion of 20% DES within the hydrogel orchestrates a transformative effect on the internal structure, aligning it more closely with the characteristics of the DES. The implication is an improved resilience to changes in solvent within the hydrogel structure, enhancing its potential as a carrier for immobilization when working with DESs. Interestingly, further analysis revealed that the highest drop in shear modulus occurred in the Alg hydrogel, which lost nearly 50% of its initial shear strength. In comparison, the 8-2 sample lost 21%, while the one infused with 20% DES only experienced a 10% decrease in shear strength after a two-week incubation in 50% DES liquid. These additional data underscore the enhanced stability conferred by the incorporation of DES within the hydrogel matrix, further supporting its utility as an immobilization carrier in DES-infused hydrogel systems.

### 3.5. Effect of Copolymer Composition on Surface Charge (Zeta Potential)

The surface charge of the copolymer samples was analyzed through zeta potential measurements; the results are shown in Table 3. The surface charge characteristics of hydrogel or copolymeric samples play a decisive role in their physicochemical properties. The alginate solution (Alg) exhibited a clearly negative zeta potential, indicating its inherent electrostatic behavior. Conversely, the 10% aqueous solution of polyvinyl alcohol (PVA) showed a zeta potential that was less than one-quarter the value of the alginate solution. Notably, upon combining PVA with alginate to form a copolymer solution, the resultant zeta potential approached that of pure alginate, indicating a change in surface charge towards the characteristics of the alginate component. This convergence in zeta potential values underscores the potential for tailoring surface charge through copolymerization.

### 3.6. Effect of DES Addition on Hydrogel Permeability

The 8-2 and 8-2-20% copolymeric hydrogels were examined for their permeability to ethyl 3-oxobutanoate. This molecule was chosen as an example of a low-water-soluble substrate for biocatalytic reduction to chiral alcohols [24], a process that can potentially be carried out using DES aqueous solutions.

**Figure 7:**
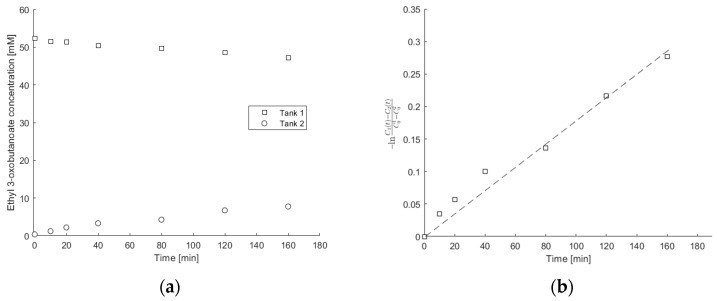
(**a**) Time variation of ethyl 3-oxobutanoate concentration in tanks 1 and 2 separated with the 8-2-10% copolymeric hydrogel prepared with 10% DES, and (**b**) variation of $-\ln\left(\frac{C_{1}(t)-C_{2}(t)}{C_{0}^{1}-C_{0}^{2}}\right)$ with time, from which the effective diffusion coefficient was calculated using Equation (5).

Concentration measurements of ethyl 3-oxobutanoate dissolved in 50% DES solutions in the two tanks shown in Figure 1, separated by an 8-2-10% copolymeric hydrogel, are shown in Figure 7a. The thickness of the hydrogel layers was measured at multiple points, as specified in Section 2.6, and ranged from 0.19 to 0.36 mm. Subsequently, the average value was calculated and employed in the determination of diffusivity coefficients. Utilizing these data and the slope derived from the fitted line in Figure 7b, the effective diffusion coefficient for ethyl 3-oxobutanoate through the copolymeric hydrogel was then calculated using Equations (5) and (6). This procedure was also conducted for other copolymeric hydrogels prepared with or without the addition of DES, as listed in Table 3.

The results of the calculated diffusion coefficients are presented in Table 4. The permeability measurements of ethyl 3-oxobutanoate indicate that the addition of 10% DES to the PVA-alginate copolymer resulted in a twofold increase in the diffusion coefficient for the selected molecule. Further addition of DES, however, led to a slight decrease in permeability, primarily attributed to an increase in shear modulus and subsequent reduction in pore size. As anticipated, the addition of PVA also contributed to a decrease in permeability, with 4% PVA exhibiting a 2.5-fold higher permeability (5.25 × 10^−10^ m^2^ s^−1^) compared to the control, while increasing the PVA content to 12% led to a small decrease in permeability.

The observed enhancement in the diffusion coefficient holds great promise for potential bioprocess applications involving immobilized biocatalysts within the hydrogel matrix. The improved mass-transport properties facilitated by DES-infused hydrogels can play a crucial role in optimizing substrate availability and promoting efficient product removal, ultimately enhancing the overall efficiency and productivity of biocatalytic reactions. Notably, the diffusion of DES-dissolved ethyl 3-oxobutanoate molecules was markedly improved within these hydrogels. This improvement is particularly significant, as it not only allows for enhanced mass transport but also ensures the preservation of superior mechanical properties even during prolonged interactions with the DES. This dual benefit positions DES-infused hydrogels as promising candidates for applications that demand both structural integrity and efficient mass transport over extended durations.

## 4. Conclusions

The optimization of hydrogels as carriers for biocatalyst immobilization has provided valuable insights. The incorporation of PVA stands out as a key factor, demonstrating a substantial enhancement in both mechanical strength and chemical resistance. Notably, the optimal concentration for this improvement was found to be 8% PVA.

Additional DES introduction in the hydrogel not only increased the mechanical properties but also improved the stability when exposed to the same environment. This dual effect suggests that the hydrogel’s resilience was enhanced by the presence of DES.

Furthermore, the addition of DES in the hydrogel proved beneficial in terms of permeability for the selected molecule when dissolved in the DES aqueous solution. This finding broadens the applicability of hydrogels, particularly in DES-assisted bioprocesses. In essence, these results illuminate the nuanced interplay between hydrogel composition and performance, paving the way for tailored applications in biocatalysis and beyond.

Notably, the versatility of DES-infused hydrogels extends to their potential as drug release carriers, as the diffusivity of molecules from these hydrogels can be finely tuned. This additional functionality enhances the scope of applications for DES hydrogels, making them promising candidates for controlled and targeted drug delivery systems.

## Figures and Tables

**Table 1:** The nomenclature and composition of the examined copolymeric hydrogels. PVA and PBA stand for polyvinyl alcohol and phenylboronic acid, respectively.

Hydrogel Abbreviation	Sodium Alginate Concentration [% (*w*/*v*)]	PVA Concentration [% (*w*/*v*)]	Crosslinking Solution
**Alg**	2	0	CaCl_2_
**2-2**	2	2	CaCl_2_ + PBA
**4-2**	2	4	CaCl_2_ + PBA
**6-2**	2	6	CaCl_2_ + PBA
**8-2**	2	8	CaCl_2_ + PBA
**10-2**	2	10	CaCl_2_ + PBA
**12-2**	2	12	CaCl_2_ + PBA

**Table 2:** Composition of the tested hydrogelswith DES addition.

Hydrogel Abbreviation	Sodium Alginate Concentration [% (*w*/*v*)]	PVA Concentration [% (*w*/*v*)]	DES Addition [% (*v*/*v*)]
**8-2-10%**	2	8	10
**8-2-20%**	2	8	20
**8-2-30%**	2	8	30
**8-2-50%**	2	8	50

**Table 3:** Zeta potential values of different copolymeric solutions.

	Mean Zeta Potential [mV]
2% Alg	−43.1
10% PVA	−10.3
4-2	−43.0
8-2	−41.9

**Table 4:** Effective diffusion coefficients for ethyl 3-oxobutanoate estimated at room temperature for various hydrogels with indicated standard deviations of two replicates (*n* = 2).

Hydrogel Abbreviation	DES Addition [% (*v*/*v*)]	Diffusion Coefficient [m^2^ s^−1^]
4-2	0	5.25 ± 0.11 × 10^−10^
8-2	0	2.14 ± 0.09 × 10^−10^
12-2	0	1.96 ± 0.1 × 10^−10^
8-2-10%	10	4.48 ± 0.13 × 10^−10^
8-2-30%	30	4.34 ± 0.16 × 10^−10^

## Data Availability

Experimental results related to this study are available at the University of Ljubljana Repository: https://repozitorij.uni-lj.si/IzpisGradiva.php?id=153126 (accessed on 9 April 2024).

## Supplementary Materials

The following supporting information can be downloaded at: https://www.mdpi.com/article/10.3390/bioengineering11040371/s1. Figure S1: The shear modulus (G) of hydrogel samples 8-2 and its homologs with PEG and BET addition (20% (*w*/*w*)). Table S1: Water content values of different hydrogel samples.
