# Supplementary material for: Tuning Mechanical Characteristics and Permeability of Alginate Hydrogel by Polyvinyl Alcohol and Deep Eutectic Solvent Addition
Source: Bioengineering (Basel). 2024 Apr 12;11(4):371. doi: 10.3390/bioengineering11040371 (PMC11048402; doi:10.3390/bioengineering11040371)
Supplement: Supplementary file 1 [file bioengineering-11-00371-s001.zip › bioengineering-2813536-supplementary.pdf]

Supplementary

Tuning Mechanical Characteristics and Permeability of Alginate Hydrogel by Polyvinyl Alcohol and Deep Eutectic Solvent Addition

Tadej Menegatti [1], Tilen Kopač [1] and Polona Žnidaršič-Plazl [1,2,*]

[1] Faculty of Chemistry and Chemical Technology, University of Ljubljana, Večna pot 113, SI-1000 Ljubljana, Slovenia; tadej.menegatti@fkkt.uni-lj.si (T.M.); tilen.kopac@fkkt.uni-lj.si (T.K.)

[2] Chair of Micro Process Engineering and Technology—COMPETE, University of Ljubljana, Večna pot 113, SI-1000 Ljubljana, Slovenia

* Correspondence: polona.znidarsic@fkkt.uni-lj.si

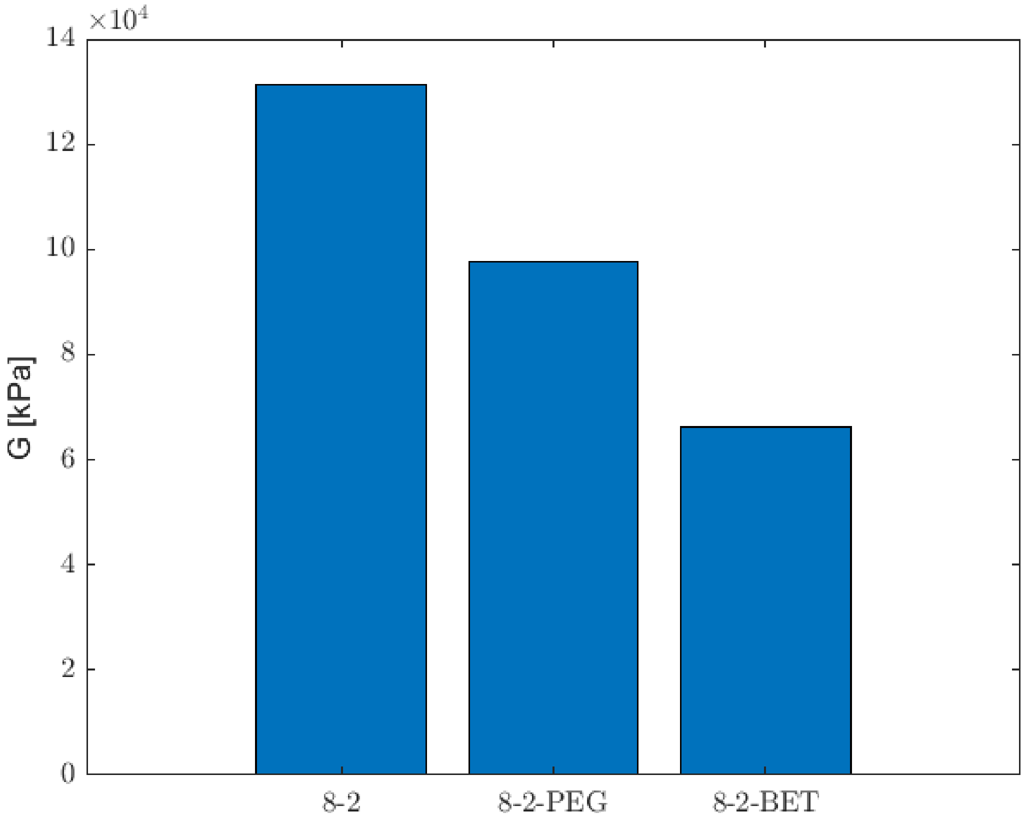

Figure S1. The shear modulus (*G*) of hydrogel samples 8-2 and its homologs with PEG and BET addition (20% (w/w)).

Table S1. Water content values of different hydrogel samples.

Hydrogel Abbreviation	Water Content [%]
Alg	89.3
4-2	85.5
8-2	83.4
12-2	81.8
8-2-30%	82.7
